# Biocompatibility of Graphene Oxide

**DOI:** 10.1007/s11671-010-9751-6

**Published:** 2010-08-21

**Authors:** Kan Wang, Jing Ruan, Hua Song, Jiali Zhang, Yan Wo, Shouwu Guo, Daxiang Cui

**Affiliations:** 1 National Key Laboratory of Nano/Micro Fabrication Technology, Key Laboratory for Thin Film and Microfabrication of Ministry of Education, Institute of Micro-Nano Science and Technology, Shanghai Jiao Tong University, 800 Dongchuan Road, Shanghai, 200240, People's Republic of China

**Keywords:** Graphene oxide, Biocompatibility, Toxicity, Cell, Mice

## Abstract

Herein, we report the effects of graphene oxides on human fibroblast cells and mice with the aim of investigating graphene oxides' biocompatibility. The graphene oxides were prepared by the modified Hummers method and characterized by high-resolution transmission electron microscope and atomic force microscopy. The human fibroblast cells were cultured with different doses of graphene oxides for day 1 to day 5. Thirty mice divided into three test groups (low, middle, high dose) and one control group were injected with 0.1, 0.25, and 0.4 mg graphene oxides, respectively, and were raised for 1 day, 7 days, and 30 days, respectively. Results showed that the water-soluble graphene oxides were successfully prepared; graphene oxides with dose less than 20 μg/mL did not exhibit toxicity to human fibroblast cells, and the dose of more than 50 μg/mL exhibits obvious cytotoxicity such as decreasing cell adhesion, inducing cell apoptosis, entering into lysosomes, mitochondrion, endoplasm, and cell nucleus. Graphene oxides under low dose (0.1 mg) and middle dose (0.25 mg) did not exhibit obvious toxicity to mice and under high dose (0.4 mg) exhibited chronic toxicity, such as 4/9 mice death and lung granuloma formation, mainly located in lung, liver, spleen, and kidney, almost could not be cleaned by kidney. In conclusion, graphene oxides exhibit dose-dependent toxicity to cells and animals, such as inducing cell apoptosis and lung granuloma formation, and cannot be cleaned by kidney. When graphene oxides are explored for in vivo applications in animal or human body, its biocompatibility must be considered.

## Introduction

In recent years, a lot of engineered nanomaterials are fabricated endlessly and investigated for their applications [1-6], and nanomaterials' biosafety has caused more and more attention from governments and scientific communities [7,8]. For example, carbon nanotubes, as the special carbon nanomaterials, have been investigated for their effects on the cells, animals and environment, and evaluated for their biosafety [9-13]. Graphene is a flat monolayer of carbon atoms tightly packed into a two-dimensional (2D) honeycomb lattice and is a basic building block for graphitic materials of all other dimensionalities with unique physical, chemical, and mechanical properties [14,15]. Graphene and graphene oxide (GO) layers have become a hotspot so far and have been actively investigated to build new composite materials [16,17]. These novel nanomaterials have great potential in applications such as electrochemical devices [18,19], energy storage [20,21] catalysis [22], adsorption of enzyme [23], cell imaging and drug delivery [24], as well as biosensors [25] However, up to date, no report is closely associated with biosafety of GO in cells or live biosystems. Here we are the first to report the effects of GO on human normal cells and mice, our results show that GO exhibits dose-dependent toxicity to cells and mice, which highly suggests that the biocompatibility of GO must be considered when the GO is applied for biomedical engineering.

## Experiments

### Synthesis and Characterization of GO

Graphene oxide (GO) was prepared from natural graphite powder by the modified Hummers method [26]. Graphite (2 g 500 mesh) and sodium nitrate (1 g) were added to a 250-mL flask at 0°C. Concentrated H_2_SO_4_ (50 mL) was then added slowly with stirring below 5°C. The mixture was stirred for 30 min and 0.3 g of KMnO_4_ was added in small portions below 10°C. The reaction mixture was stirred for an additional 30 min and 7 g of KMnO_4_ was added to the mixture respectively over 1 h below 20°C. After the temperature of the mixture warmed to 35 ± 3°C and stirred for 2 h, 90 mL of water was slowly dripped into the paste, causing an increase in temperature to 70°C and the diluted suspension was stirred at this temperature for another 15 min. Then, it was further treated with a mixture of H_2_O_2_ (30%, 7 mL) and water (55 mL). The resulting suspension turned bright yellow, and the warm suspension (about 40°C) was filtered, resulting in a yellow–brown filter cake. The cake was washed for three times with a warm solution of 3% aqueous HCl (150 mL), followed by drying at 40°C for 24 h in vacuo. Finally, the GO was obtained by ultrasonication of as-made graphite oxide in water for 1 h. The resulting homogeneous yellow–brown dispersion was tested to be stable until now. GO was analyzed by the FT-IR technique showed the presence of GO, using Fourier transform infrared (EQUINOX 55, Bruker, Germany) spectrometer. Furthermore, the structure and the texture of GO were observed by transmission electron microscopy (TEM) and AFM (Figure 2), which are JEOL JEM-2010 TEM and Nanoscope MultiMode SPM (Veeco, American).

### Effects of GO on Human Fibroblast Cells

In order to investigate the cytotoxicity of GO in vitro, we chose human fibroblast cell (HDF) as the target cells to evaluate the cell viability and proliferation by CCK8 assays. Every well in the 96-well plate was planted 5,000 cells and incubated in a humidified 5% CO_2_ balanced air incubator at 37°C for 24 h. Except from control wells, the contents in the remaining wells were added into medium with GO and the final concentrations were 5, 10, 20, 50, 100 μg/mL, respectively, next continued to culture from day 1 to day 5, we measured the absorbency using the Thermo multiskan MK3 ELISA plate reader according to the protocol of CCK8 assay and calculated the survival rate of cells. The survival rate of cells can be calculated by the following equation:

(1) $$\text{Survival rate of cells }\left(\%\right)=A_{570}\left(\text{sample}\right)/A_{570}\left(\text{control}\right)\times 100$$

where *A*_570_(sample) is absorbance intensity at 570 nm in the presence of GO, and *A*_570_(control) is absorbance intensity at 570 nm in the absence of GO.

The cell attachment assay was performed as previously described [27]. Essentially, 6-well plates were coated with fibrinogen (5 μg/mL) and vitronectin (1.5 μg/mL) in DPBS. Cells were harvested, washed three times with serum-free minimal essential medium with Eargle's salt and resuspended in attachment solution (calcium- and magnesium-free Hanks' balanced salt solution, 20 mM HEPES, 1 mg/mL heat-inactivated BSA, 1 mM CaCl_2_ and 1 mM MgCl_2_). Cells (1 × 10^4^) were added to each well and allowed to culture for 1–5 days at 37°C in a humidified 5% CO_2_ incubator. These plates of respective 5, 10, 20, 50 and 100 μg/mL GO-treated cells were cultured for 1–5 days, and 1 control plate was set up (1 × 10^4^ cells were added into each well, which was treated with 0.5% DMSO vehicle and allowed to culture for 1–5 days at 37°C in a humidified 5% CO_2_ incubator) and were centrifuged for 10 min at the speed of 4,000 rpm. Unattached cells were washed with Hanks' balanced salt solution. The number of remaining attached cells after centrifugation was quantified spectrophotometrically at 405 nm in triplicate [28]. Cell adhesion ability (%) = the number of GO-treated adhesive cells/the number of control adhesive cells.

Human fibroblast cells (HDF) were treated for 5 days with different concentrations of GO: 5, 10, 20, 50, and 100 μg/mL. After incubation, cells were lysed in protein lysis buffer. Equal amounts of sample lysate were separated by sodium dodecylsulfatepolyacrylamide gel electrophoresis (SDS–PAGE) and electrophoretically transferred onto polyvinylidene difluoride (PVDF) membranes (Millipore). The membrane was blocked with 0.1% BSA in TBST buffer and incubated overnight at 4°C with specific primary antibodies. Subsequently, the membrane was washed with TBST buffer and incubated with horseradish peroxidase-conjugated secondary antibodies. Enhanced chemiluminescence kits were used (Amersham, ECL kits) [29]. In order to confirm whether GO can stimulate HDF cells secrete small molecular proteins, HDF cells were cultured for 5 days in essential medium without 10% fetal calf serum with the aim of excluding mistaking fetal calf serum proteins as secreted small molecular proteins.

HDF was treated with 20 μg/mL GO and cultured in a humidified 5% CO_2_ balanced air incubator at 37°C for 24 h, then fixed cells with 2.5% glutaraldehyde solution and embedded with epoxy resin, finally made the ultrathin cell specimen and observed the specimen with TEM.

### Effects of GO on Mice

All animal experiments performed in compliance with the local ethics committee. Kunming mice (female, 28–30 g, 4–5 weeks old) were obtained from the Shanghai LAC Laboratory Animal Co. Ltd., Chinese Academy of Sciences (Shanghai, China) and housed in positive-pressure air-conditioned units (25°C, 50% relative humidity) on a 12:12-h light/dark cycle. The mice were allowed to acclimate at this facility for 1 week before being used in the experiment. Each mouse was exposed to the GO suspension via a single tail vein injection. The mice were killed at 1, 7, and 30 days post exposure, and their organs, including heart, liver, spleen, stomach, kidneys, lungs and brain, were collected. For conventional histology, tissues were collected immediately after killing, fixed in 10% formaldehyde, embedded in paraffin, cut into 20-μm-thick section, stained with hematoxylin and eosin, and examined by light microscopy. Three mice were used for negative control.

### Statistical Analysis

Each experiment was repeated three times in duplicate. The results were presented as mean ± SD.

Statistical differences were evaluated using the *t*-test and considered significance at *P* < 0.05.

## Results and Discussion

### Characterization of GO

**Figure 1:**
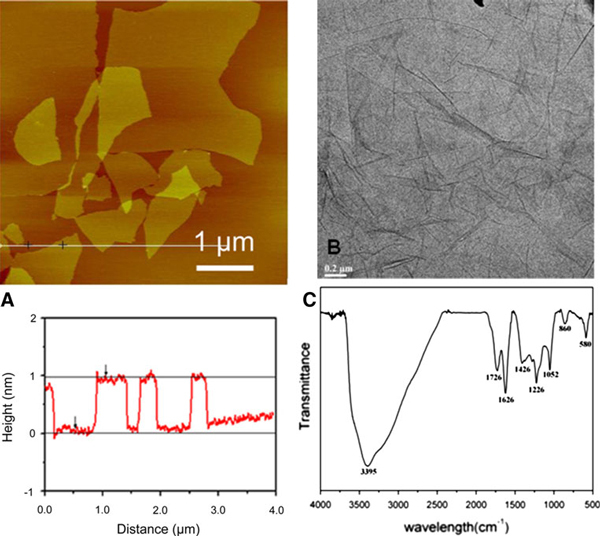
**Characterization of graphene oxides: a AFM image of GO, b TEM image of GO, c FT-IR spectrum of GO**.

The prepared GO was water-soluble, black, and dispersed well. As shown in Figure 1, the spectrum of FT-IR of GO showed that the peak at 3,395 cm^-1^ attributes to O–H stretching vibration, the peak at 1,726 cm^-1^ attributes to C=O stretching vibration, the peak at 1,426 cm^-1^ attributes to deformation of O–H, the peak at 1,226 cm^-1^ attributes to vibration of C–O (epoxy), and the peak at 1,052 cm^-1^ attributes to vibration of C–O (alkoxy). AFM image of GO showed that the GO sheet is flat and smooth, and the height of GO sheet is about 1 nm, indicating the mono-layer GO sheet was successfully prepared. The TEM image of GO also confirmed the GO existed in the sheet-like shapes. Therefore, water-soluble graphene oxides were successfully prepared.

### Effects of GO on Human Fibroblast (HDF) Cells

**Figure 2:**
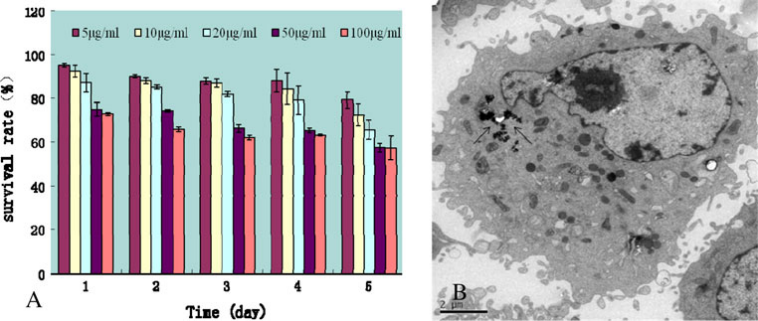
**Effects of GO on human fibroblast cells: a the HDF survival rate at different concentrations of GO and different culture time, b TEM picture of location of GO inside HDF cells as shown by the *arrows***.

Regarding the effects of GO on HDF cells, as shown in Figure 2a, GO below 20 μg/mL exhibited low cytotoxicity, the cell survival rate is more than 80%, above 50 μg/mL exhibited obvious cytotoxicity such as decreasing cell survival rate, inducing cell floating and cell apoptosis. As the cell culture day increased, the survival rate of cells decreased correspondingly, highly dependent on GO dose and culture time. As shown in Figure 2b, GO was indeed internalized by cells and mainly located inside cytoplasm such as lysosomes, mitochondrion, and endoplasm. We also observed that, as the culture time increased, the amount of GO inside HDF cells increased accordingly, and lot of GO appeared as black dots scattered in the cell cytoplasm around cell nuclear, a few GO located inside nucleus.

### Effects of GO on Cell Adhesive Proteins

**Figure 3:**
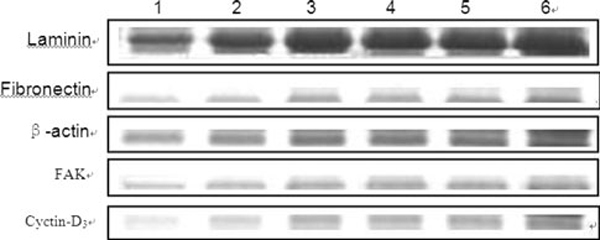
**Western blot analysis of adhesion proteins in HDF cells cultured with different concentrations of GO for 5 days**. *Lanes 1*–*6* show the expression levels of proteins in HDF cells treated with GO with the following concentrations: 100, 50, 20, 10, 5, 0 μg/mL, respectively

**Figure 4:**
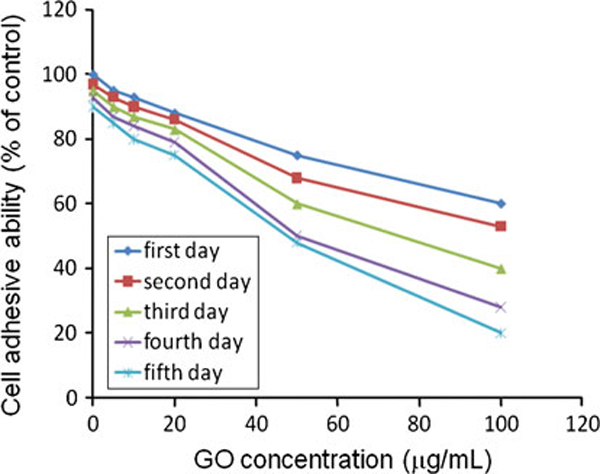
**GO-treated HDF cell adhesion ability measured by centrifugation method**. The percentage of adhesive cells decreased with the increase in GO concentration and culture time

The adhesive ability of GO-treated HDF cells can be evaluated with the ratio of GO-treated adhesive cell number to the control adhesive cell number after centrifuge. As shown in Figure 3, the cell adhesive ability decreased markedly with the increase in GO concentration and culture time. Western blot results showed that, comparing with normal cells, the expression levels of laminin, fibronectin, FAK, and cell cycle protein cyclin D3 in the HDF cells treated with GO were markedly decreased, and their expression levels in HDF cells cultured with GO decreased gradually as the amount of GO increased as shown in Figure 4, the β-actin protein expression remained unchanged in each case. There is a significant difference (*P* < 0.05) between GO-treated groups and normal control group.

### Effects of GO on Cell Morphology

**Figure 5:**
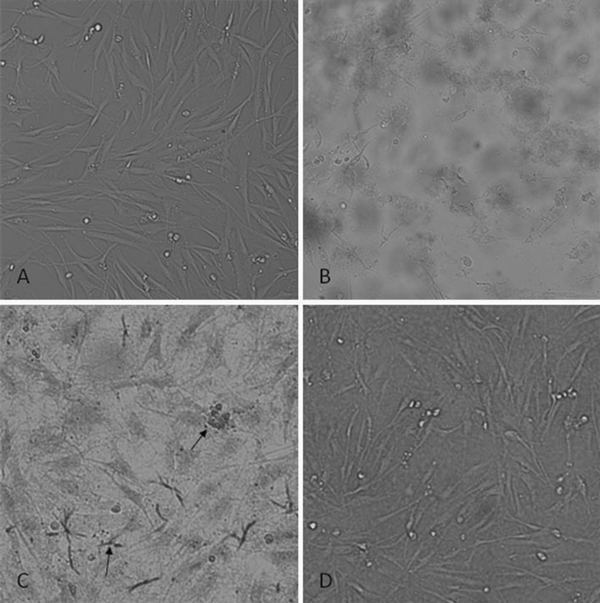
**Apoptosis of HDF cells induced by GO: a normal HDF cells, showing normal morphological cells, b morphological changes of HDF cells cultured with 100 μg/mL GO for 1 day, cells appear inner vacuole and apoptotic bodies showing apoptotic characteristics, c morphology of HDF cells cultured with 20 μg/mL GO for 3 days, showing cell have unclear boundary, membrane vesicles and fragmentation, the arrow showing apoptotic cells formed nodular structure encapsulating GO, d morphology of HDF cells cultured with 5 μg/mL GO for 5 days, showing normal cell morphology**.

Microscopic observation of GO-treated HDF cells showed that, compared with control cells as shown in Figure 5a, some HDF cells rounded up, detached from the culture plates and displayed morphological changes characteristic of apoptosis after 24 h of incubation as the dose of GO in the medium reached 100 μg/mL as shown in Figure 5b, HDF cells cultured with 20 μg/mL GO for 72 h exhibited features characteristic of apoptosis such as membrane vesicles, fragmentation and unclear cell boundary, apoptotic cells formed nodular structure encapsulating GO as shown in Figure 5c. HDF cells cultured with 5 μg/mL GO for 100 h showed normal cell morphology except to rough cell surface as shown in Figure 5d.

### Effects of GO on Lifespan of Mice

Regarding the effects of GO on mice, we used tail vein injection pathway to evaluate the in vivo toxicity. The mice were injected with 0 mg (control group), 0.1 mg (low dose group, LD), 0.25 mg (medium dose group, MD), and 0.4 mg (high dose group, HD) GO per mouse. After 1 day, 1 week, and 1 month exposure, the mice were killed by the method of cervical vertebra displace, and then used histopathology to evaluate inflammation degree of the mouse organs.

Injection dose of GO at 0.1 and 0.25 mg per mouse did not cause mortality of exposed mice, and showed no obvious clinical toxic signs, and the body weight of treated mice accordingly increased with the raise time increasing. However, 4 of 9 mice treated with 0.4 mg per mouse died (1/3 in the 1-day group, 1/3 in the 7-day group and 2/3 in the 30-day group). All deaths occurred 1–7 days after injection of the GO. The deaths were generally preceded by lethargy, inactivity, and body-weight losses. Histopathology of lung tissues showed that major airways of four mice were mechanically blocked by the GO conglomeration, which led to suffocation in 15% of the GO-exposed mice, and was not evidence of pulmonary toxicity of GO. In addition, the survival mice treated with 0.4 mg of GO for 24 h appeared weakness and lost 10% of body weights within first week, this symptoms disappeared after one week, as evidenced by subsequent normal eating behavior and weight increase.

### Effects of GO on Important Organs

**Figure 6:**
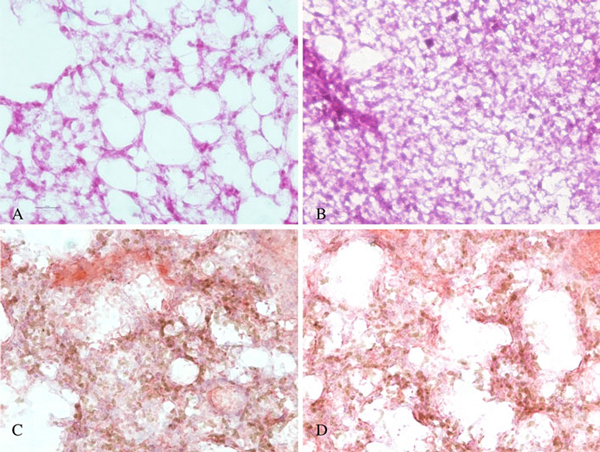
**The light micrograph of lung tissues about rats exposed to different dose graphene sheets for 7 days: a control: 0 mg, b 0.1 mg, c 0.25 mg, d 0.4 mg (magnification = ×200)**.

We also investigated the effects of GO on organs of mice. We learn from the pathology and light micrograph that the GO accumulations were primarily in the lungs, liver, and spleen. There were obvious chronic toxicity responses occurring in the lungs and liver after tail vein injection. Histopathological analysis revealed that pulmonary exposures to GO produced a dose-dependent lung inflammatory response characterized by neutrophils and foamy alveolar macrophage accumulation. Figure 6 showed the light micrograph of lung tissues from mice exposed to different doses of GO for 7 days, clearly showed that the treated mice exhibited a dose-dependent series of granulomas. With the increase in GO dose, the toxicity reaction of the lung of mice becomes more and more severe. For example, GO induced dose-dependent epithelioid granulomas and, in some cases, interstitial inflammation in the mice. Large amount of inflammation cells was infiltrated in lung alveolus interstitium; the alveolar septa became thicker and some lung alveoli were cracked.

**Figure 7:**
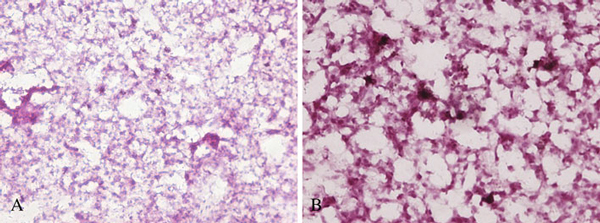
**The light micrograph of lung tissues from mice exposed to GO of 0.1 mg at different exposure time: a 7 days, b 30 days (magnification = ×200)**.

Figure 7 is the light micrograph of lung tissues from mice exposed to GO of 0.1 mg by tail vein injection at different exposure time. The early development of lesions was first observed at 7 days, wherein the lesions surrounded the GO, and this was associated with a nonuniform, diffuse pattern of GO particulate deposition in the lung. Subsequently, at 30 days, a diffuse pattern of multifocal macrophage-containing granulomas was presented. It was interesting to note that few lesions existed in some lobes, while other lobes contained several granulomatous lesions. This was likely due to the nonuniform deposition pattern following GO instillation. At higher magnification, one could discern the discrete multifocal mononuclear granulomas centered around the GO.

**Figure 8:**
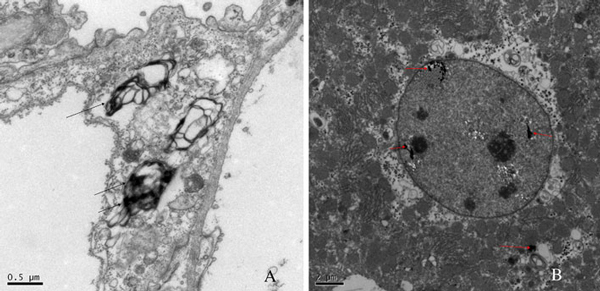
**TEM images of the ultrastructural features of the lung and live tissue: a lung tissue, b live tissue, *arrows* point to GO**.

In order to observe the high accumulation levels and to assess the biological effects of GO in mice organs, ultrathin sections were prepared from the harvested mice lungs and liver for TEM imaging. Figure 8 showed the TEM images of the ultrastructural features of the lung and live tissues exposed to GO. The GO still remained in the lungs after 1 month, some in capillary vessel and some in cytoplasmic vacuoles of lung tissues (Figure 8a). There were a lot of inflammation cells appeared in the wall of lung vacuole, such as multinuclear giant cells and acidophilic cells. The ultrastructural features of most cells appeared pathological changes. As shown in Figure 8b, the GO was found to be entrapped in the phagosome of a hepatic macrophage.

Regarding the biodistribution of GO in mice, as we observed, GO mainly located in lung, liver and spleen, no GO was found in the brain tissues, which highly suggests that GO cannot get through blood–brain barrier. Few GO was observed in kidney of mice, which highly suggests that GO is very difficult to be exited out by pathway of kidney, we speculate that GO mainly is expelled out by liver secretion into bile tract system.

### The Potential Mechanism of Effects of GO on Human Cells

As mentioned earlier, we clearly observed that graphene oxides with dose of less than 20 μg/mL did not exhibit toxicity to human fibroblast cells, and the dose of more than 50 μg/mL exhibits obvious cytotoxicity such as decreasing cell adhesion, inducing cell apoptosis, entering into lysosomes, mitochondrion, endoplasm, and cell nucleus. Similar phenomena were also observed in other cell lines such as human gastric cancer MGC803, human breast cancer MCF-7, MDA-MB-435, and liver cancer HepG2 cell lines (data not shown), which highly suggest that GO exhibits obvious toxicity to human normal cells or tumor cells. According to our results, we suggest the possible mechanism of GO's cytotoxicity as follows: GO in medium attach to the surface of human cells, providing a stimuli signal to the cells. The signal is transduced inside the cells and the nucleus, leading to down-regulation of adhesion-associated genes and corresponding adhesive proteins, resulting in decrease in cell adhesion and causing cells to detach, float, and shrink in size. At the same time, GO enters into cytoplasm by endocytosis pathway, mainly located in the lysosomes, mitochondrion, endoplasm and cell nucleus, may disturb the course of cell energy metabolism and gene transcription and translation, and finally result in cell apoptosis or death.

### The Possible Mechanism of Effects of GO on Mice

As we observed, graphene oxides under low dose (0.1 mg) and middle dose (0.25 mg) did not exhibit obvious toxicity to mice, under high dose (0.4 mg) exhibited chronic toxicity such as 4/9 mice death and lung granuloma formation, mainly located in lung, liver, spleen and kidney, almost could not be cleaned by kidney. The possible mechanism of effects of GO on mice is suggested as follows: when GO enters into mouse body by vessel injection, directly enter into blood circulation system, as one kind of foreign body, which should be recognized and tracked by immune cells, GO quickly distributes into lung, liver, spleen, and kidney, but cannot enter into brain due to blood–brain barrier. When GO enters into lung tissues, provides a stimulating signal to lung cells, under synergic action of lung cells and immune cells, GO is captured and wrapped by immune cells, finally results in lung granuloma formation, GO in liver, spleen, and kidney may cause corresponding inflammation. Because of flake-shapes of GO, GO is very difficult to be kicked out by kidney, thus stay in liver, spleen, and kidney for long term, at the lower dose, these organs such as liver, spleen, and kidney can tolerate and maintain their normal function, at higher dose, lot of GO in liver, spleen, and kidney can damage the balance and badly affect the function of these organs, result in failure of organ function and death of mice. Regarding the effects of immune cells on GO in vivo in mice, the possible mechanism is not clarified well and still needs further research.

## Conclusion

In conclusion, our primary studies have indicated that GO could produce cytotoxicity in dose- and time-dependent means, and can enter into cytoplasm and nucleus, decreasing cell adhesion, inducing cell floating and apoptosis. GO can enter into lung tissues and stop there and induce lung inflammation and subsequent granulomas highly dependent on injected dose. Exposures to GO may induce severe cytotoxicity and lung diseases. It should be the first report. Although GO has been investigated for biomedical applications such as cell imaging and drug delivery [30-35], because of GO's long-term stay in kidney and being very difficult to be cleaned by kidney, therefore, GO may not own good application prospect in human body. How to decrease or abolish the toxicity of GO is still a challengeable task for in vivo biomedical application. Further work will focus on investigating the possible mechanism of interaction between GO and immune cells in human body or mice.
